# Supplementary material for: Learning curve and surgical outcome of robotic assisted colorectal surgery with ERAS program
Source: Sci Rep. 2022 Nov 29;12:20566. doi: 10.1038/s41598-022-24665-w (PMC9709162; doi:10.1038/s41598-022-24665-w)
Supplement: Supplementary file 1 — Supplementary Information 1. [file 41598_2022_24665_MOESM1_ESM.zip › LSC Raw data-Ñ[▒K/090/N_ñw╝╨░O▒Kñσ.pdf]

W55 / W55

臺中榮民總醫院
手術室護理記錄

[redacted]

基本資料

- 室別： 二醫手術室-31室，手術科別： CRS，手術類別： 常規，日期： 2020/09/10
- 進入時間： 1310 ； 麻醉開始時間： 1315 ； Time Out時間： 1407 ； 劃刀時間： 1410 ； 手術結束時間： 1855 送出時間： 1900 ；
- 麻醉方式： GE

  診斷： Malignant tumor of RS COLON WITH BLADDER INVASION

  手術名稱： 1.Low anterior resection with colo-anal anastomosis2.Laparoscopy3.4.

  手術局部用藥： NIL

  術前預防性抗生素：
  - 抗生素名稱一： Veterin 1GM IVD(由麻醉科護理人員給予)
  - 時間： 1340
  - 上次簽章: 吳雅華
  - 抗生素名稱二： Veterin 1GM IVD(由麻醉科護理人員給予)
  - 時間： 1750
  - 記錄者： 陳昱穎

  ，追加預防性抗生素：
- 手術醫師： 蔣鋒帆，黃昆霖，楊曜榮，楊捷儒
- 麻醉醫師： VS 賴慧卿
-
  - 刷手護士A ： 林宜旻，時間： 1310-1500，接替刷手一： 吳若綺，時間： 1500-1700，接替刷手二： 陳昱穎，時間： 1700-1730
  - 流動護士A ： 吳雅華，時間： 1310-1500，接替流動一： 洪紹瓊，時間： 1500-1700，接替流動二： 彭馨慧，時間： 1700-1730
- 更多接替及流動護士： 接替刷手三： 吳若綺，時間： 1730-1900，接替流動三： 吳明柔，時間： 1730-1800，接替流動四： 陳昱穎，時間： 1800-1900

皮膚狀況

A-V Shunt： 無

術前： 完整

記錄者： 上次簽章: 林宜旻

術後： 完整

記錄者： 上次簽章: 陳昱穎

手術準備

手術臥式 單一手術臥式

-
  - 手術臥式： 截石臥式
  - 使用附件： 矽膠枕頭，腳架
  - 皮膚準備： 10%Aq Betadine，Hibiscrub，2% Chlorhexidine
  - 電燒： 使用電燒: 電極片位置： 其它： 迴路電極墊，記錄者： 上次簽章: 林宜旻

止血帶T： 無

植入物

- 植入物名稱/號碼/數量/部位： surgiwrap/壹/小/in pelvic

，記錄者： 上次簽章: 陳昱穎

標本送檢

- 細菌培養 無
- 細胞學 無
- 冰凍切片 無
- 病理標本

組織名稱一： rectum

,

組織名稱二： IMA root

,

組織名稱三： distal cutend

- 截肢 無

標本交付 標本： 共? 袋 ，標本： 零 盒 ，標本： 零 瓶 ，細胞學： 零 管 ，Culture Tube： 零 管 ，被交付醫師： CR 楊曙榮，

- 記錄者： 上次簽章: 陳昱穎
- 時間： 1855

傷口術後敷料

傷口敷料：

傷口敷料： 無

- 請於圖上標示部位：

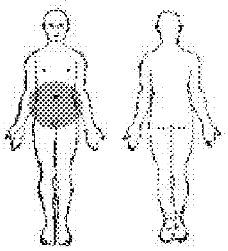

腹腔鏡切口

- 傷口敷料： MEPORE

導管

導管	天數	導管資訊	插入日期	插入時間	更換日期	拔除日期	拔除時間	拔除原因	預計拔除/更換	預計拔除/更換日期
A-line(1)	1	號碼:20 G; 部位:前臂; 象限:LU;	2020/09/10	1340						
Foley(1)	1	號碼:16 Fr.; 部位:膀胱;	2020/09/10	1400						
Rod,Stoma Supporting (1)	1	號碼:壹 ; 部位:終段迴腸; 註記:右腹;	2020/09/10	1855						
Penrose Drain(1)	1	號碼:12 mm.; 部位:骨盆腔;	2020/09/10	1700						
Chemo Port(1)	155	號碼:8.0 Fr.; 部位:左鎖骨下靜脈; 註記:Polysite;	2020/04/09	1315						

用物計數

- 手術中出血量： minimal c.c.
- 手術中尿量： 500 c.c.
-
    - 其它： 無

用物計數： 多切口同時縫合

- 1.器械： 正確 ，2.縫針： 正確 ，3.刀片 ： 正確 ，4.紗布： 正確 ，5.腹部墊： 未使用 ，6.紗球： 未使用 ，7.PEANUTS ： 未使用 ，8.紗條： 未使用 ，9.絲帶： 未使用 ，10.針頭： 未使用 ，11.Vessel loop： 未使用 ，12.Boots： 未使用 ，13.Jelco： 未使用 ，14.棉條： 未使用 ，
    - 第一次：
        - 刷手護士 ： 吳若綺
        - 流動護士： 上次簽章: 吳明柔
        - 時間： 1730

,

-
    - 第二次：
        - 刷手護士 ： 吳若綺
        - 流動護士： 上次簽章: 吳明柔
        - 時間： 1745

,

- 第三次：
  - 刷手護士 ： 吳若綺
  - 流動護士： 上次簽章: 陳昱穎
  - 時間： 1800

備註

備註： NIL

- 記錄者： 上次簽章: 洪紹瓊
- 時間： 1518

填寫護理人員 陳昱穎

正式摘要

W55 / W55

doctorRank:B
